# Strength Evolution of Concrete with Different Coal Gangue and Fly Ash Contents

**DOI:** 10.3390/ma19194185

**Published:** 2026-09-30

**Authors:** Weili Li, Guang Yang, Fenghui Li, Changan Xie, Yunhai Cheng

**Affiliations:** 1 Hefei Design and Research Institute of Coal Industry Co., Ltd., Hefei 230000, China; 15855403969@163.com; 2 School of Mining Engineering, Anhui University of Science and Technology, Huainan 232001, China; 3 School of Mechanical and Electrical Engineering, Huainan Normal University, Huainan 232000, China; ahlifenghui@163.com; 4 National Engineering Laboratory of Coalmine Backfilling Mining, Shandong University of Science and Technology, Taian 271000, China; xca10001@163.com (C.X.); chengyunhai2005@163.com (Y.C.)

**Keywords:** coal gangue, fly ash, concrete, strength evolution rules, failure characteristics

## Abstract

To explore the application feasibility of coal gangue and fly ash in underground floor concrete, this study adopted fly ash to partially replace cement and coal gangue to partially replace natural aggregates and prepared concrete specimens with various curing ages to investigate their strength evolution rules and long-term performance of floor concrete. The mix proportion was determined via physical and chemical tests on raw materials, as well as slump and compressive strength tests. The strength, stress–strain, and failure characteristics of concrete at different ages were analyzed through uniaxial compression and Brazilian splitting methods. The research results show that the optimal mix ratio is a water–binder ratio of 0.46, fly ash substitution rate of 30%, coal gangue substitution rate of 40%, and slump ≥160 mm. It meets the needs of underground pumping construction, and its 28-day compressive strength meets the requirements for C30 concrete. The concrete strength increases rapidly at early ages and grows slowly in the later stage. The compressive strength of the optimal group rises to 43.03 MPa at 70 days, an increase of 29.2% compared with its 28-day strength. The compressive strength of groups with high fly ash content is generally low. The failure mode of the specimen is mainly multi-crack breakage, with interlaced cracks and strong penetration. The research findings facilitate the large-scale disposal and utilization of coal gangue and fly ash solid wastes, cut down the consumption of natural aggregates and cement, and satisfy the development demands of green mining.

## 1. Introduction

Gangue is a solid waste produced during coal mining and selection, and fly ash is produced in the combustion and utilization process. Both are main solid wastes. Among them, the annual output of coal gangue reaches 825 million tons [1], which not only occupies massive land resources, but also causes adverse impacts on the environment. Therefore, effective treatment and application of them have become an important issue facing the coal industry [2].

Fly ash can improve the mobility of concrete mixture, reduce cement amount, reduce carbon emissions, and reduce hydration heat hazards [3].

Ritu [4] and Kannur B [5] believe that concrete and other fields are important application scenarios for fly ash. Etcheverry et al. [6] used low-calcium fly ash to replace over 50% of cement at room temperature and ensured that the 28-day compressive strength reached the standard. Zeng et al. [7] prepared concrete via cement activation and alkali activation processes using fly ash in excess of 75% as the main raw material. The single fly ash system has shortcomings including low early strength, slow hydration at low temperatures, and increased brittleness under high-dosage conditions. Accordingly, scholars worldwide have gradually conducted research on composite modification with multiple solid wastes. Materials such as slag [8], silica fume [9,10], metakaolin [11], zeolite [12], recycled concrete powder [13], limestone powder [14], phosphogypsum [15], recycled glass powder [16], and plastic waste [17] are adopted to synergize with or compound with fly ash [18]. The synergistic hydration of active components compensates for the disadvantages of single fly ash incorporation.

Using coal gangue as aggregate to replace traditional gravel for concrete preparation can not only reduce the demand for natural mineral resources but also prevent the ecological damage caused by the exploitation of stone and river sand and promote the rational recycling of coal gangue resources [19].

Su Yuxiang [20] tests show that replacing part of natural aggregates with coal gangue within a reasonable range will not reduce the compressive strength of concrete. Guo Qiushuo, Ren X et al. [21,22] conducted a study on using gangue instead of gravel as coarse and fine aggregate, which showed that when the replacement rate of coal gangue coarse aggregate exceeds 30%, the concrete strength decreases significantly. Song et al. [23] pointed out that adding fiber, fly ash, nanomaterials, etc. can improve the problems of insufficient strength and easy cracking of gangue concrete. Li Wen [24] believes that reasonable gradation of aggregates with different particle sizes can effectively fill internal voids of materials, thereby reducing the occurrence and development of damage. Gao B et al. [25] further discussed the mechanism of spontaneous combustion of gangue and believed that coal gangue possesses high pozzolanic activity after spontaneous combustion. Pan Cheng [26] proposed an elastic–plastic constitutive relationship theory based on coal gangue concrete, believing that numerous micropores inside coal gangue can dissipate external force energy and inhibit crack propagation. Qu et al. [27] studied the influence of the synergistic threshold of red mud, fly ash, and coal gangue on material properties. The balance between strength and volume stability was studied through testing indicators such as compressive strength and dry shrinkage. Yang et al. [28] studied the modification of coal gangue–fly ash geopolymer concrete by basalt fiber. Adding fibers with a trace volume fraction can inhibit the propagation of microcracks, greatly improve the flexural strength, and adapt to a high-humidity underground environment. Li et al. [29] studied alkali-stimulated fly ash-slag-based coal gangue permeable concrete. After alkali-stimulated hydration reaction and fiber cracking resistance, the strength loss rate after 180 days of sulfate erosion was only 8.2%, providing a solution for water-rich and high-sulfate mining areas. Murtaza et al. [30], with the core goals of improving the durability of high-grade self-compacting concrete and resource utilization of multiple solid waste, established a four-component solid waste blending system consisting of fly ash, coal gangue powder, cement kiln dust and recycled concrete powder to study the long-term durability of materials.

Wang et al. [31] investigated the underground application of coal gangue–fly ash concrete with fly ash content below 20%. Appropriate fly ash can improve the workability of concrete, while the strength decreases with the increase in coal gangue content. Liu et al. [32] studied the frost resistance durability and pore evolution of fly ash coal gangue concrete. High content of coal gangue accelerates frost–thaw deterioration. SEM observations reveal severe crack propagation and a weak interfacial transition zone in specimens with high coal gangue replacement. Niu et al. [33] established a quadratic regression model for strength development of coal gangue concrete modified by fly ash–metakaolin. The model shows high significance with *p* < 0.001 and the coefficient of determination R^2^ > 0.95, indicating reliable prediction accuracy.

Underground floor paving puts forward requirements for material strength and durability. Based on actual engineering needs, fly ash was used to replace part of cement, and coal gangue to replace part of natural aggregate, and concrete samples with different curing ages are prepared. Through uniaxial compression and splitting tensile test, the mechanical properties and damage characteristics of samples under different substitution rates are analyzed.

## 2. Experimental Material Characteristics

### 2.1. Chemical Composition of Coal Gangue

The coal gangue used in this test was crushed and screened, and 200–300 mesh coal gangue powder was sampled and tested by an X-ray diffractometer (XRD, (Rigaku Corporation, Takatsuki, Osaka Prefecture, Japan)). The photograph of coal gangue is shown in Figure 1. The results are shown in Figure 2.

Coal gangue is mainly composed of quartz, calcite, and anorthite. Its primary chemical components are Al_2_O_3_ and SiO_2_, which together account for over 50% of the total mass.

### 2.2. Physical Characteristics of Coal Gangue

#### 2.2.1. Particle Grading

For pumped concrete used for underground floor bedding, the maximum nominal particle size of coarse aggregate shall not exceed 1/4 of the minimum section size of structural members. In the test, for a cubic specimen with a side length of 100 mm, the maximum particle size of the aggregate needs to be less than 25 mm. In order to improve the fluidity, the maximum particle size of the aggregate is selected to be 20 mm. The grading of gangue particles is shown in Table 1. The grading of gangue particles is shown in Figure 3.

It can be seen from Table 1 that the particle size range of 5–10 mm accounts for 39.3%, the particle size range of 10–16 mm accounts for 32.2%, and the particle size of 16–20 mm accounts for 16.5%. Combined with the requirements for the supporting performance of the coarse aggregate skeleton in underground floor laying projects, the maximum particle size of the coarse aggregate is determined to be 20 mm. In order to facilitate subsequent mix ratio design and experimental research, coal gangue is divided into two categories: large-particle-size gangue (10–20 mm) and small-particle-size gangue (5–10 mm) according to particle size range, with the proportions of each level being 67% and 33% respectively.

For natural gravel, the maximum particle size of coarse aggregate is also set to 20 mm. In order to facilitate subsequent mix design and experimental research, the particle size and proportion are consistent with those of coal gangue.

#### 2.2.2. Physical Properties of Coarse Aggregate

The physical properties of natural gravel and coal gangue are shown in Table 2.

The moisture content, needle-shaped particle content, and crushing value of coal gangue are obviously higher than those of natural gravel, and the differences of other performance indicators between the two aggregates are insignificant.

### 2.3. Fly Ash

The fly ash used in this test came from a thermoelectric power station in Huainan, and its main components are as follows in Table 3, and the X-ray diffraction analysis is shown in Figure 4. The main components are SiO_2_ and Al_2_O_3_.

A 45 mm square-hole sieve was adopted for sieving, and the sieve residue was 9.2%. According to the fineness test results, this fly ash is classified as Class I fly ash.

### 2.4. Cement

Cement is used as the main binding material of concrete. P·O 42.5 Conch ordinary Portland cement was used for this experiment. Its main mineral compositions are tricalcium silicate (C_3_S), dicalcium silicate (C_2_S), and tricalcium aluminate (C_3_A), of which tricalcium silicate accounts for 40–60% of cement clinker. Dicalcium silicate accounts for 20–30% of the clinker. See Table 4 below for the main components of the cement sample. The phase analysis results completed through X-ray diffraction test are as shown in Figure 5.

### 2.5. Other Materials

(1) Natural Yellow River sand is used for the fine aggregate of the concrete in this test.

(2) Natural gravel is used for coarse aggregate, and the apparent properties have been reflected in the comparison between the front section and coal gangue.

(3) The water used for concrete mixing is tap water from the municipal water supply system of Huainan City.

(4) The admixture is made of polycarboxylate superplasticizer, and the water reduction rate is 25%.

## 3. Experimental Plan Design

The superplasticizer was added at a dosage of 3% by mass of cement. The mixture ratio of the obtained C30 fly ash concrete is shown in Table 5.

Aiming at the research on coal gangue–fly ash concrete, part of the aggregate was replaced with gangue–fly ash concrete based on the mass mix ratio of fly ash concrete. The experiment was designed according to the response surface design method to ensure that the water amount and the total amount of coarse aggregate remained unchanged, and three variables were selected: water–binder ratio, fly ash replacement rate and coal gangue replacement rate. In order to ensure the strength, the water–binder ratio was set to 0.44, 0.46, and 0.48; the fly ash replacement rate for cement (by mass) was set as 20%, 30%, and 40%; the coal gangue replacement rate for gravel (by mass) was set to 30%, 40%, and 50%. The designed mix ratio of coal gangue and fly ash concrete is as follows in Table 6.

Specimens were prepared in accordance with the designed mix proportions and then cured. First pour natural gravel, coal gangue, and sand into a forced mixer and dry mix for 30 s to mix the aggregate evenly. Add cement and fly ash and dry mix for 60 s to ensure that the cementitious material covers the aggregate surface. Dissolve the additive in water according to the designed dosage, then add about 80% water and water reducing agent, and stir for 90 s; finally add the remaining 20% water and continue to stir for 120 s until the mixture has uniform color and no lumps. The total stirring time is 5 min.

Remove the mold 24 h after curing and forming. Then placed in a standard curing environment (temperature 20 ± 2 °C, relative humidity ≥ 95%) for 7 d, 14 d, 21 d, and 28 d.

## 4. Strength Evolution of Concrete with Different Fly Ash and Coal Gangue Parameters

After curing for 24 h, the test pieces are removed and numbered as shown in Figure 6. The concrete specimens are cured to the specified age and then taken out.

The bearing surfaces of specimens were polished to control the flatness within 0.05 mm. The experiment was carried out using a TYJ-1500M rock mechanics testing machine (Nantong Huaxing Petroleum Instrument Co., Ltd., Nantong, Jiangsu, China), and the displacement and loading rate of 100 mm cubic specimens were 0.5~0.6 mm/min.

The average strength was calculated from three specimens in each group; if the strength of a single specimen deviates from the average by more than 15%, the average value of the remaining values is taken after excluding this value. If two specimens show deviations exceeding 15%, the test group is deemed invalid.

For underlaying concrete, the slump needs to reach more than 160 mm, and its fluctuation shall be strictly controlled. In order to simultaneously present the mechanical properties and construction fluidity of concrete, based on the compressive strength data measured in the test, a nested histogram of compressive strength and a dot line diagram of measured slump data are drawn, as shown in Figure 7.

(1) Slump analysis

The slump of concrete is made of iron sheet slump barrel (100 mm × 200 mm × 300 mm).

Each test group meets the workability requirements of mining concrete. Compared with test group No. 3 and No. 12, the slump decreased from 184 mm to 161 mm, with a decrease of 12.5%, indicating that the slump of concrete will gradually increase with the increase in the water–binder ratio variable and the fly ash replacement cement ratio variable.

(2) Analysis of uniaxial test results

The 7d strength range is 14.68~22.61 MPa, which is about 53~57% of the 28d strength; the 14d strength range is 19.37~28.39 MPa, which is about 69~77% of the 28d strength; the 21d strength range is 24.18~34.53 MPa, which is about 85~92% of the 28d strength; the 28-day strength range is 30.68~39.40 MPa. All groups meet the C30 strength requirements (≥30 MPa), and FGC-12 reaches the C40 level.

The compressive experimental results were fitted and analyzed, and a quadratic regression response function model was obtained between the compressive strength of concrete at each age and the water–binder ratio X_1_, the ratio of fly ash replacing cement X_2_, and the ratio of coal gangue replacing coarse aggregate X_3_.

$$Y^{*}=\beta_{0}+\sum_{i=1}^{n}\beta_{i}X_{i}+\sum_{i=1}^{n}\beta_{ii}X_{i}^{2}+\sum_{i=1}^{n}\sum_{i=1}^{n}\beta_{ij}X_{i}X_{j}$$

Y*—equation response value; β_0_—constant term; β_i_, β_ii_, β_ij_—correlation regression coefficients; X_i_, X_j_—independent variables; n—number of influencing factors.

Compressive strength at 7d age:

$$Y_{7d}^{*}=17.36-0.95X_{1}-2.72X_{2}-0.48X_{3}+0.40X_{1}X_{2}+0.14X_{1}X_{3}+0.09X_{2}X_{3}+0.46X_{1}^{2}+0.43X_{2}^{2}+0.08X_{3}^{2}$$

Compressive strength at 14d age:

$$Y_{14d}^{*}=22.38-1.04X_{1}-3.15X_{2}-0.59X_{3}+0.33X_{1}X_{2}+0.22X_{1}X_{3}+0.21X_{2}X_{3}+0.59X_{1}^{2}+0.58X_{2}^{2}-0.07X_{3}^{2}$$

Compressive strength at 21d age:

$$Y_{21d}^{*}=27.38-1.36X_{1}-3.60X_{2}-0.91X_{3}+0.73X_{1}X_{2}+0.29X_{1}X_{3}+0.61X_{2}X_{3}+0.43X_{1}^{2}+0.82X_{2}^{2}+0.07X_{3}^{2}$$

Compressive strength at 28d age:

$$Y_{28d}^{*}=33.27-1.35X_{1}-2.38X_{2}-0.93X_{3}+0.84X_{1}X_{2}+0.64X_{1}X_{3}+0.38X_{2}X_{3}+0.45X_{1}^{2}+0.79X_{2}^{2}-0.62X_{3}^{2}$$

In order to verify the reliability of the functional model, variance analysis was performed on the response results of the above functional model, and the variance analysis results are shown in the table. It can be seen from the table that the *p* < 0.001 and the F values for all age models are between 35.91 and 86.94 (as shown in Table 7), indicating that the regression model is significant and can effectively describe the relationship between independent variables and compressive strength.

The 28-day strength is often used as the standard compressive strength of concrete. Combining the response surface and contour area changes, the influence of various variables on concrete strength can be obtained.

The relationship between water–binder ratio, fly ash replacement rate and strength is shown in Figure 8a. As the water–binder ratio increases and the fly ash replacement rate increases, the compressive strength decreases significantly, which is consistent with the negative correlation conclusion of the linear term. The 28-day strength of concrete increases with the decrease in water–binder ratio and first stabilizes and then decreases significantly with the increase in fly ash replacement rate.

The relationship between water–binder ratio, coal gangue substitution rate, and strength is shown in Figure 8b. The slope of the response surface is relatively moderate, and the effect of coal gangue substitution rate on strength is weaker than that of fly ash substitution rate.

The relationship between fly ash substitution rate, coal gangue substitution rate, and strength is shown in Figure 8c. Both factors have negative correlation effects, and the fly ash substitution rate has a greater influence weight; the 28d strength of concrete increases with the decrease in fly ash substitution rate and fluctuates steadily first and then gradually decreases with the increase in coal gangue substitution rate.

## 5. Long-Age Strength Evolution Rule of Concrete with Different Fly Ash and Coal Gangue Parameters

Underground underfloor concrete needs to meet the requirements of compressive strength, engineering economy and long-term stability at the same time. The 70-day age is the key node for the stabilization of the hydration reaction of pozzolanic active materials, which can effectively characterize the long-term mechanical characteristics of concrete. Therefore, FGC-2, FGC-9, FGC-8, and the control group FC-2 were selected to carry out long-age mechanical properties research. See Table 8 for the mix ratio of each group. Cubic specimens with a size of 100 mm × 100 mm × 100 mm were prepared and cured in standard curing environment until 70 days.

After curing, prepare cylindrical specimens with a size of 50 mm × 50 mm.

As shown in Figure 9, the tensile strength was measured using the Brazilian splitting method, and the loading was carried out at a constant speed at a rate of 0.5~0.6 mm/min. During the loading process, the deformation of the specimen was observed, and the maximum failure load was recorded until the specimen was completely split.

7d is the early strength of concrete, 28d is the standard age, and 70d is the long age. The specimens after failure are shown in Figure 10 below.

The uniaxial data at different ages and long-age tensile strength are shown in Table 9, and the change trend of compressive strength and tensile strength are shown in Figure 11.

(1) Compressive strength analysis

7d strength: FC-2 (22.36 MPa) > FGC-2 (21.06 MPa) > FGC-9 (17.12 MPa) > FGC-8 (14.86 MPa), and the compressive strength of high fly ash substitution groups is generally low. At this time, the failure mode of the specimen is mainly multi-crack breakage, with interlaced cracks and strong penetration.

28d strength: FC-2 (39.29 MPa) > FGC-2 (36.97 MPa) > FGC-9 (33.31 MPa) > FGC-8 (30.68 MPa). The strength still decreases with the increase in coal gangue substitution rate, and the corresponding failure form shows that the number of cracks decreases and becomes more concentrated.

70d strength: FC-2 (46.88 MPa) > FGC-2 (46.51 MPa) > FGC-9 (43.03 MPa) > FGC-8 (39.36 MPa), and the overall trend decreases with the increase in the substitution rate of fly ash and coal gangue. Among them, the strength of FGC-2 is very similar to that of FC-2 in the group without coal gangue being mixed; the strength of each group in the FGC series decreases with the substitution rate of coal gangue rising from 30% to 50%, which confirms that coal gangue replacing natural coarse aggregate has a negative correlation effect on long-age strength. In terms of failure mode, the 70-day test specimens were mainly fragmented in overall blocks, with few cracks and weak penetration; FC-2 and FGC-2 still retained large and complete aggregate blocks after failure, and the interface bonding properties were excellent; FGC-8 the aggregate spalling is relatively noticeable.

(2) Tensile strength analysis

70d tensile strength: FC-2 (4.11 MPa) > FGC-2 (2.96 MPa) > FGC-9 (2.69 MPa) > FGC-8 (2.56 MPa) is consistent with the rule of 70d compressive strength.

Mining floor concrete is frequently subjected to tramcar rolling. In addition to reaching the specified compressive strength, it also needs to have a certain tensile toughness to resist the tensile stress caused by the deformation of the surrounding rock. The design splitting tensile strength of C30 concrete should be ≥2.59 MPa, which can be met by FC-2, FGC-2, and FGC-9. From the perspective of comprehensive engineering applicability, the FGC9 group has a fly ash substitution rate of 30%, and a coal gangue substitution rate of 40%. The 70-day compressive strength reaches 43.03 MPa, which meets the design requirements of underground heavy-duty floors, and the splitting tensile performance is also stable; at the same time, the content of coal gangue and fly ash is higher, and the benefits of solid waste resource utilization are outstanding. FGC9 group is a better practical ratio choice for projects.

(3) Characteristics of stress–strain curves

After uniaxial compression tests at different ages, the stress–strain curves of FC-2, FGC-2, FGC-9, and FGC-8 specimens are shown in Figure 12 below.

The uniaxial compression stress–strain curves of each group of concrete at different ages all show typical three-stage characteristics: elastic stage, plastic yielding stage and failure stage. However, due to the influence of coal gangue substitution rate, fly ash substitution rate and curing age, the characteristics of each stage are significantly different.

When the curing age is the same, the proportion of coal gangue replacing natural gravel has a significant impact on the stress evolution characteristics of concrete during uniaxial compression. When the substitution rate of coal gangue is 30%, there is no significant difference in the stress growth rate and peak stress compared with the control group without coal gangue, which confirms that the bearing capacity of concrete is not significantly weakened under this substitution ratio, but the rate of stress change decreases if the substitution rate of coal gangue is continued to increase.

It can be seen from Table 10 that the elastic moduli increase rapidly to slowly with age, with a significant increase from 7 d to 28 d, and a decrease from 28 d to 70 d. The area under the curve is used to represent the energy absorption capacity of concrete, and the order of the 70-day age is FGC-8 > FGC-9 > FGC-2 > FC-2. The higher the ratio of coal gangue replacing coarse aggregate, the fuller the curve, and the stronger the energy absorption capacity, the more suitable it is for mining floor laying scenarios with large deformation of surrounding rock.

## 6. Conclusions

Focusing on the resource utilization of coal gangue and fly ash and the performance requirements of underground floor paving materials, this paper systematically studies the ratio design and mechanical characteristics of coal gangue and fly ash concrete through laboratory experiments. The main conclusions are as follows:

(1) The optimal mix proportion of coal gangue–fly ash concrete was determined. The water–binder ratio is 0.46, the fly ash substitution rate is 30%, and the coal gangue substitution rate is 40%. Under this ratio, the concrete slump is ≥160 mm, which meets the requirements for underground pumping. The 28-day compressive strength is 33.31 MPa, meeting the C30 strength requirements.

(2) The coupling effects of fly ash and coal gangue replacement rates on concrete performance and failure characteristics were clarified. Concrete strength rises rapidly at early ages and then grows slowly, and both admixtures show negative correlations with compressive strength.

(3) The compressive strength of FGC-9 group reached 43.03 MPa at a long age of 70 days, which was 29.2% higher than its 28-day strength. The secondary hydration effect of fly ash and coal gangue during the long age can effectively make up for the strength disadvantage caused by high substitution rate; A positive correlation exists between splitting tensile strength and compressive strength.

(4) The stress–strain curve shows three stages of elasticity, plastic yield, and failure. The specimens show better ductility and energy dissipation capabilities at high solid waste replacement rates.

This research realizes the win–win of economic and environmental benefits. This technology can realize the large-scale utilization of coal gangue and fly ash, reduce the amount of natural aggregate and cement, and has theoretical and practical value for green mine construction.

## Figures and Tables

**Figure 1:**
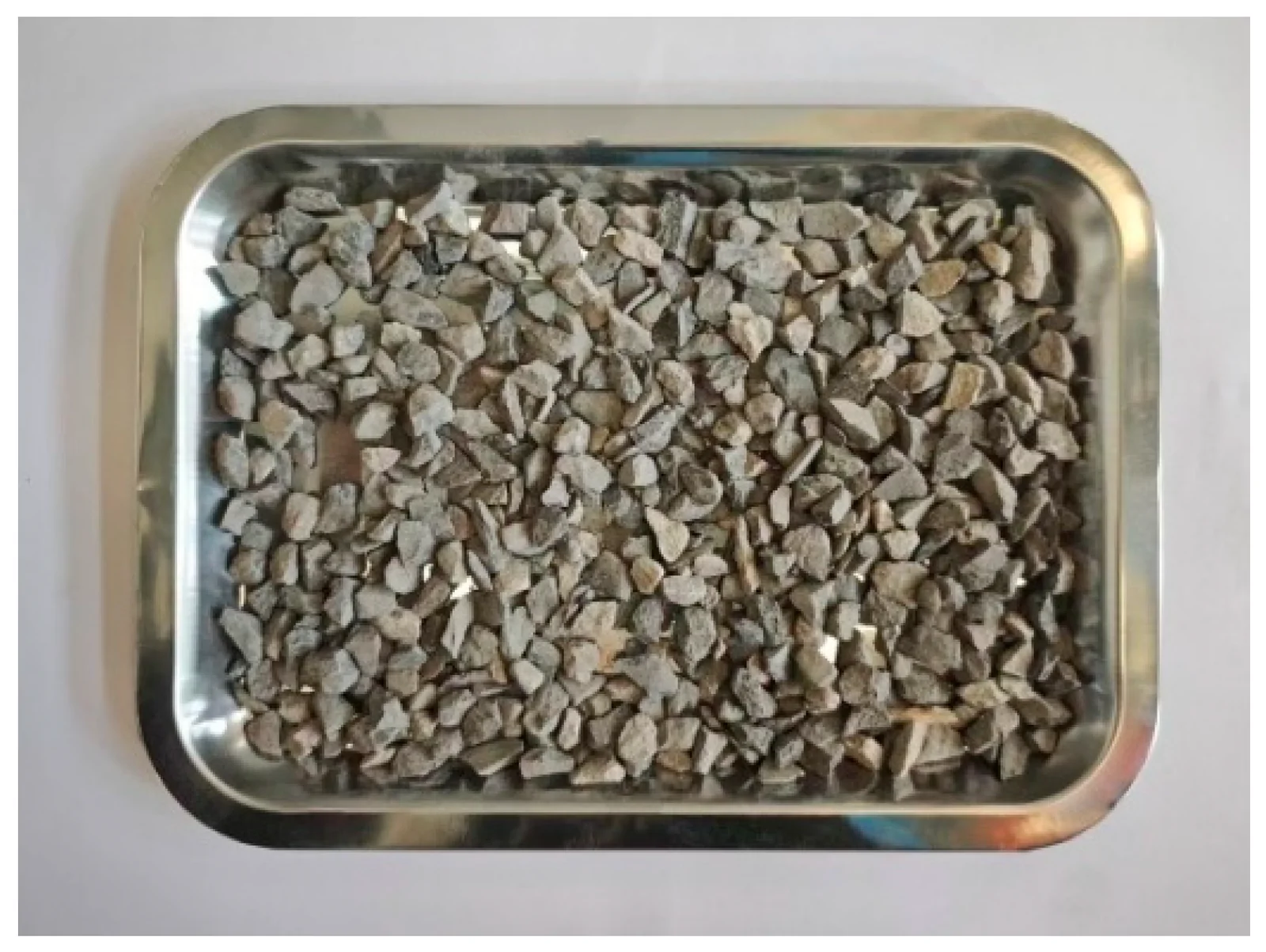
Coal gangue.

**Figure 2:**
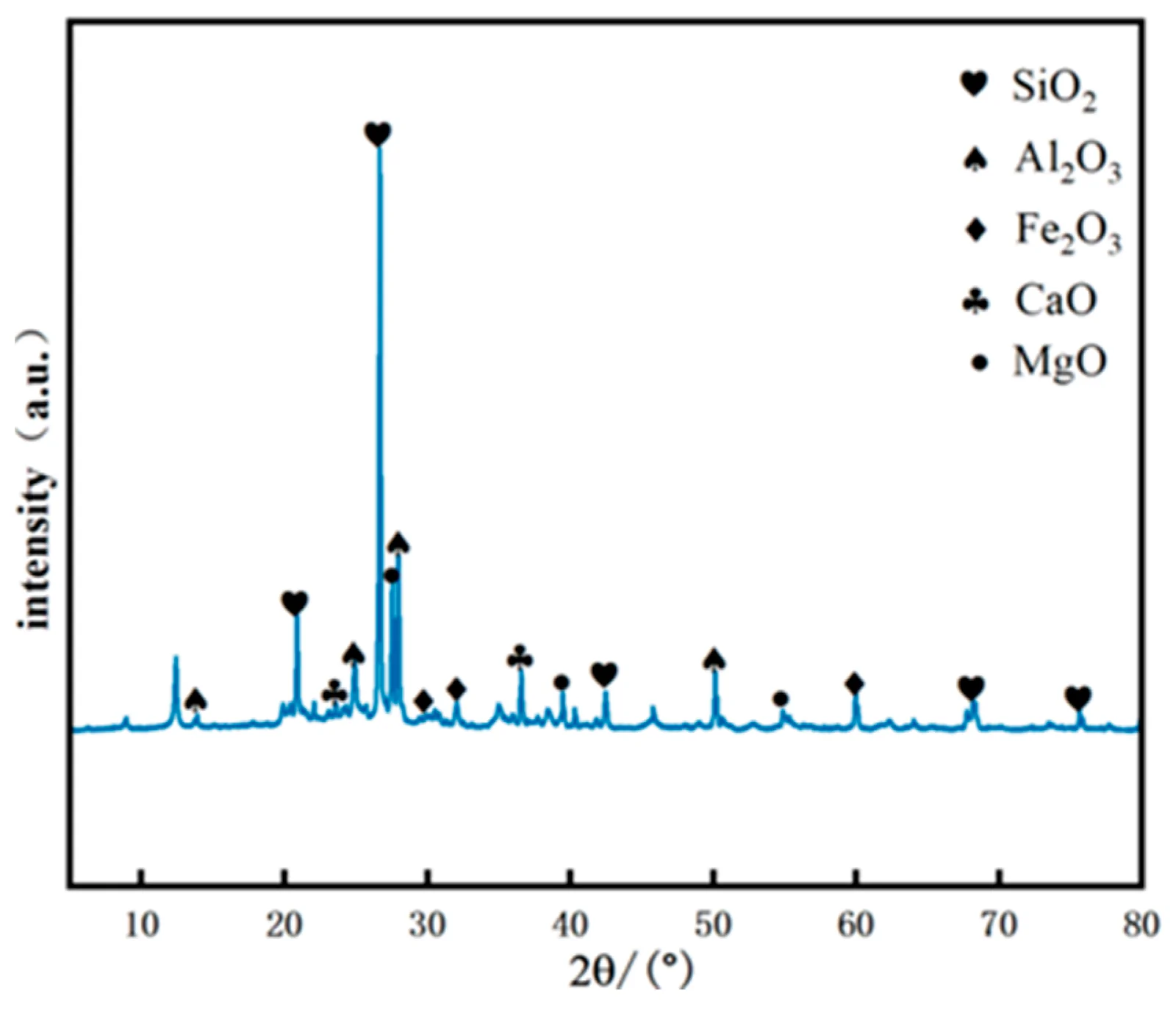
XRD pattern of coal gangue.

**Figure 3:**
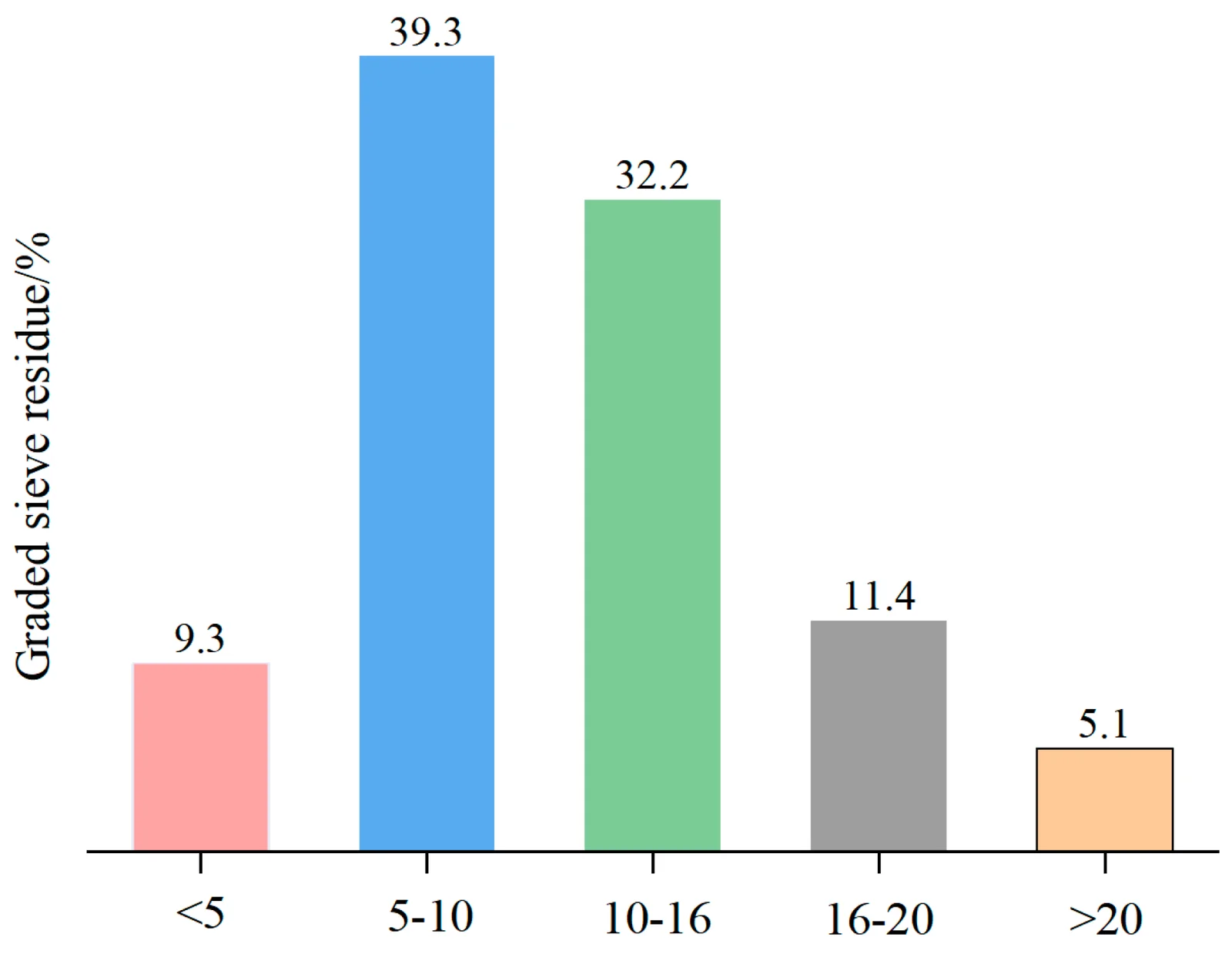
Particle size distribution of coal gangue.

**Figure 4:**
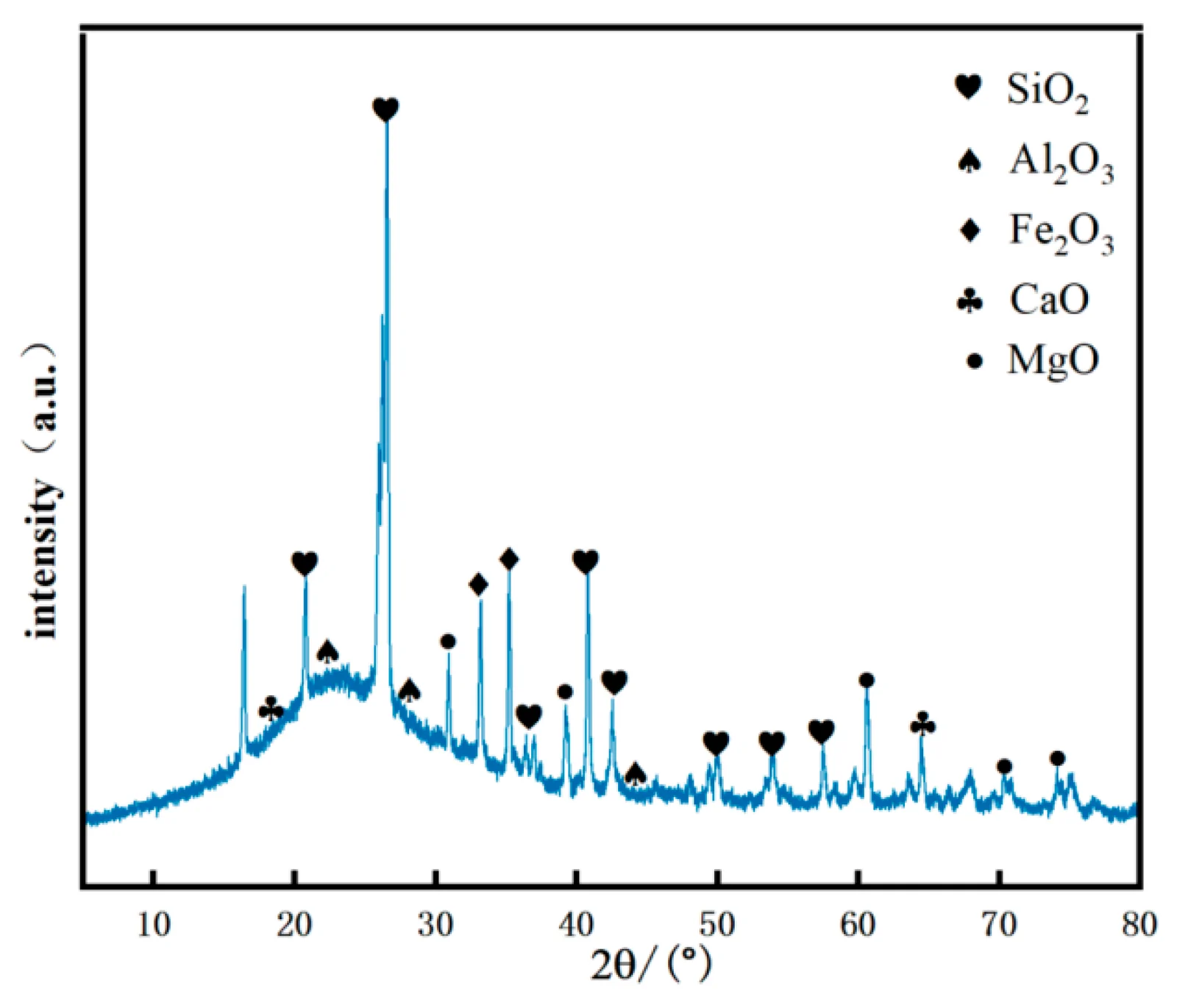
XRD pattern of fly ash.

**Figure 5:**
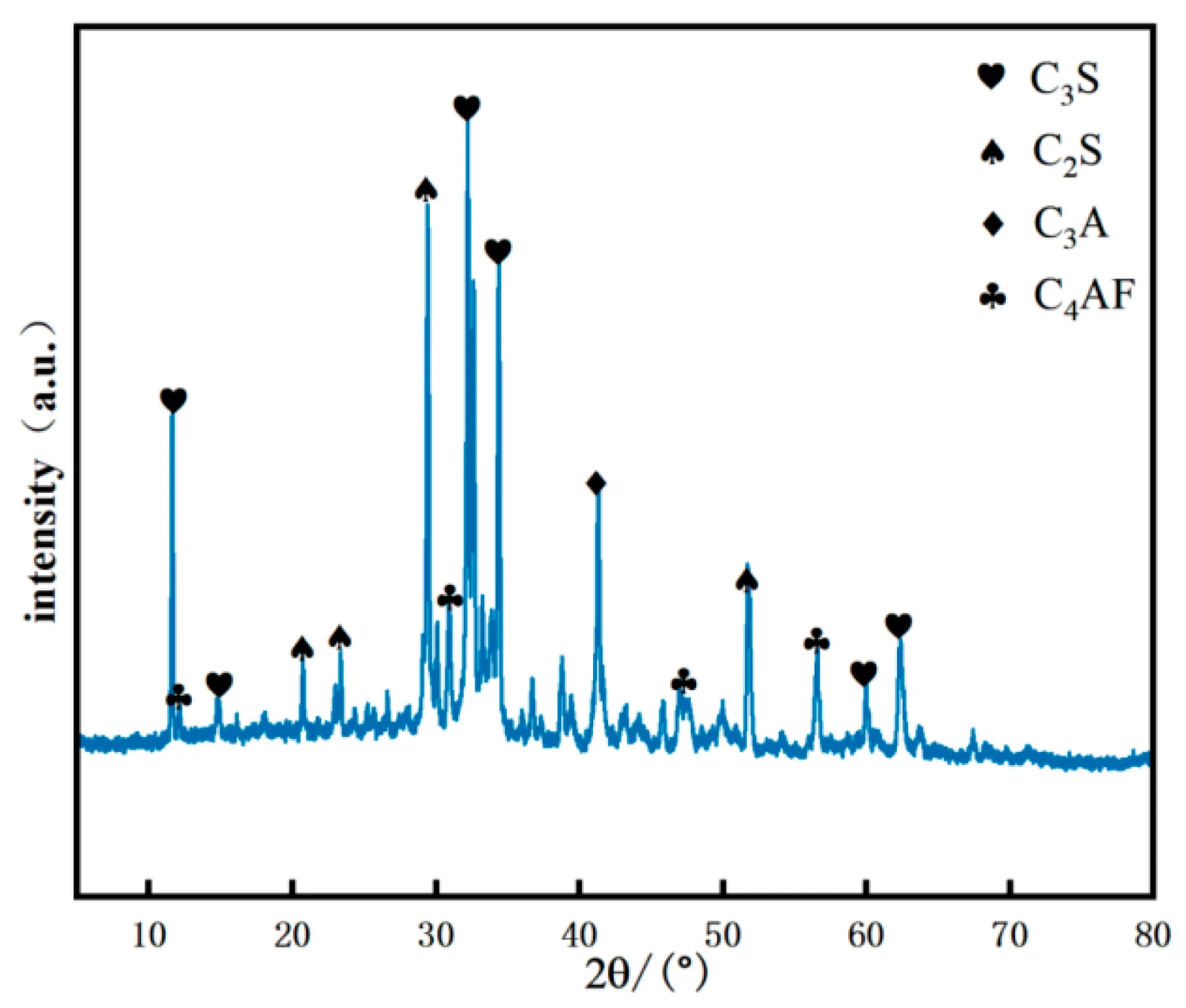
XRD pattern of cement.

**Figure 6:**
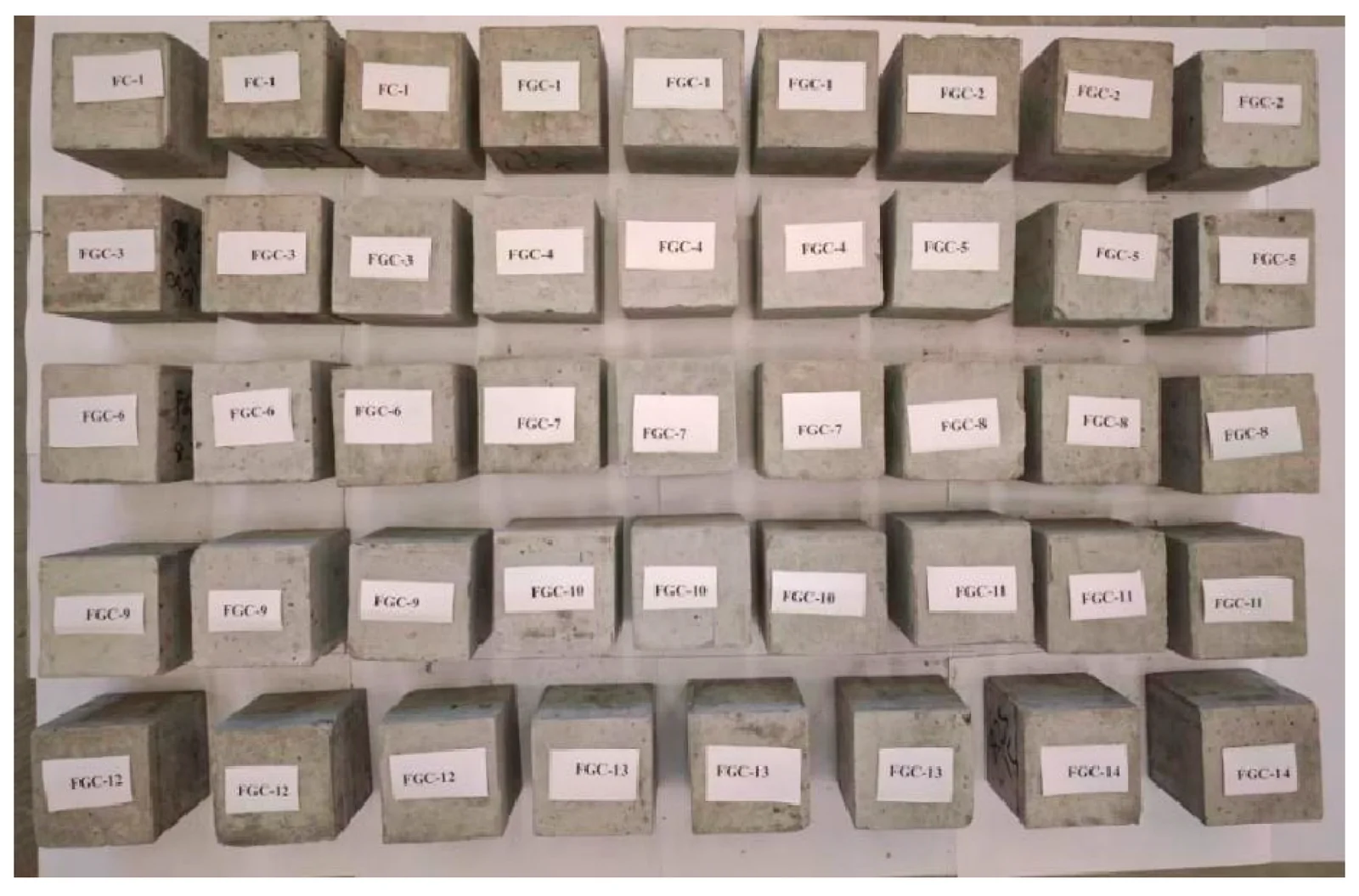
Partial concrete specimens image.

**Figure 7:**
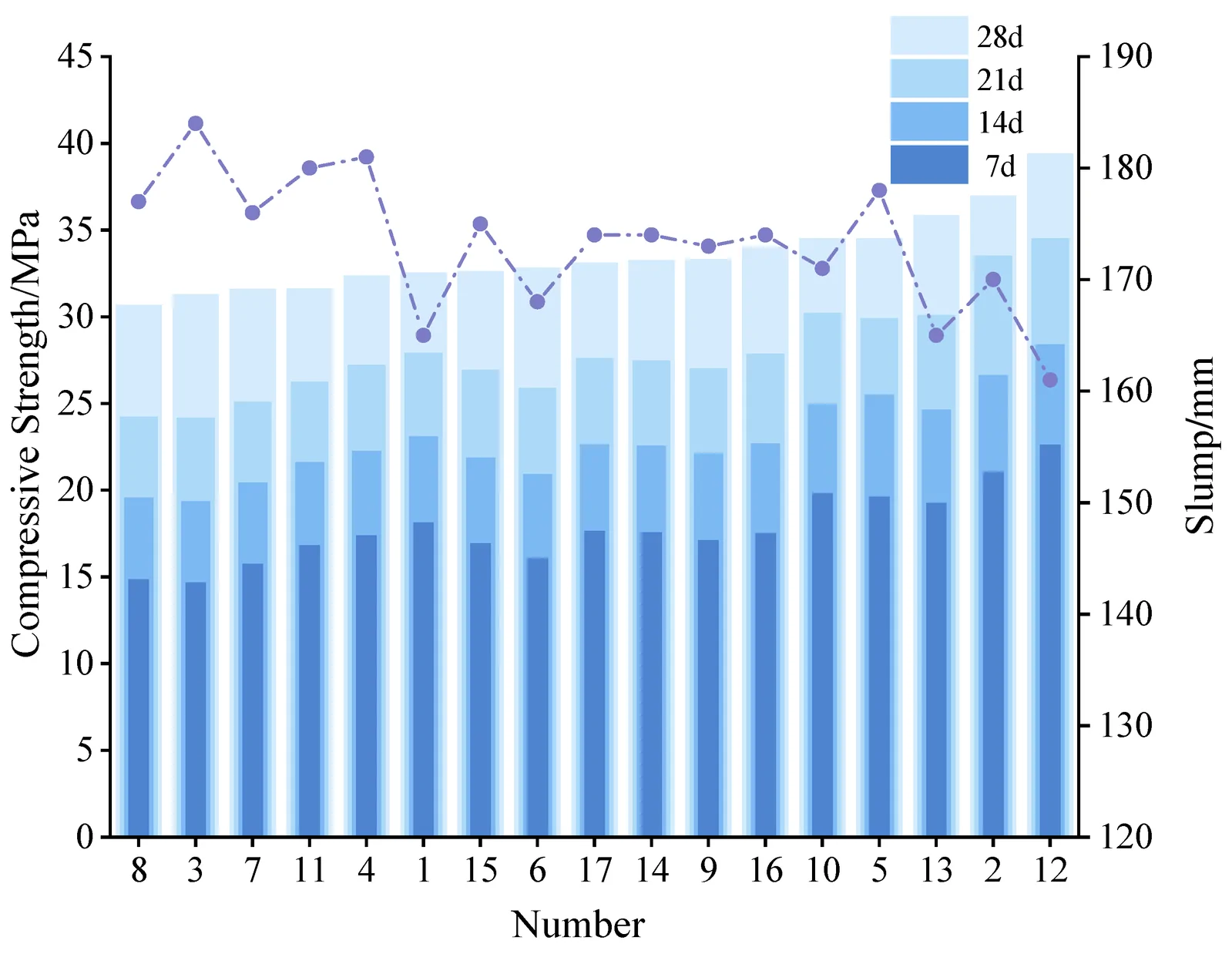
Nested bar chart of compressive strength and dot–line chart of slump.

**Figure 8:**
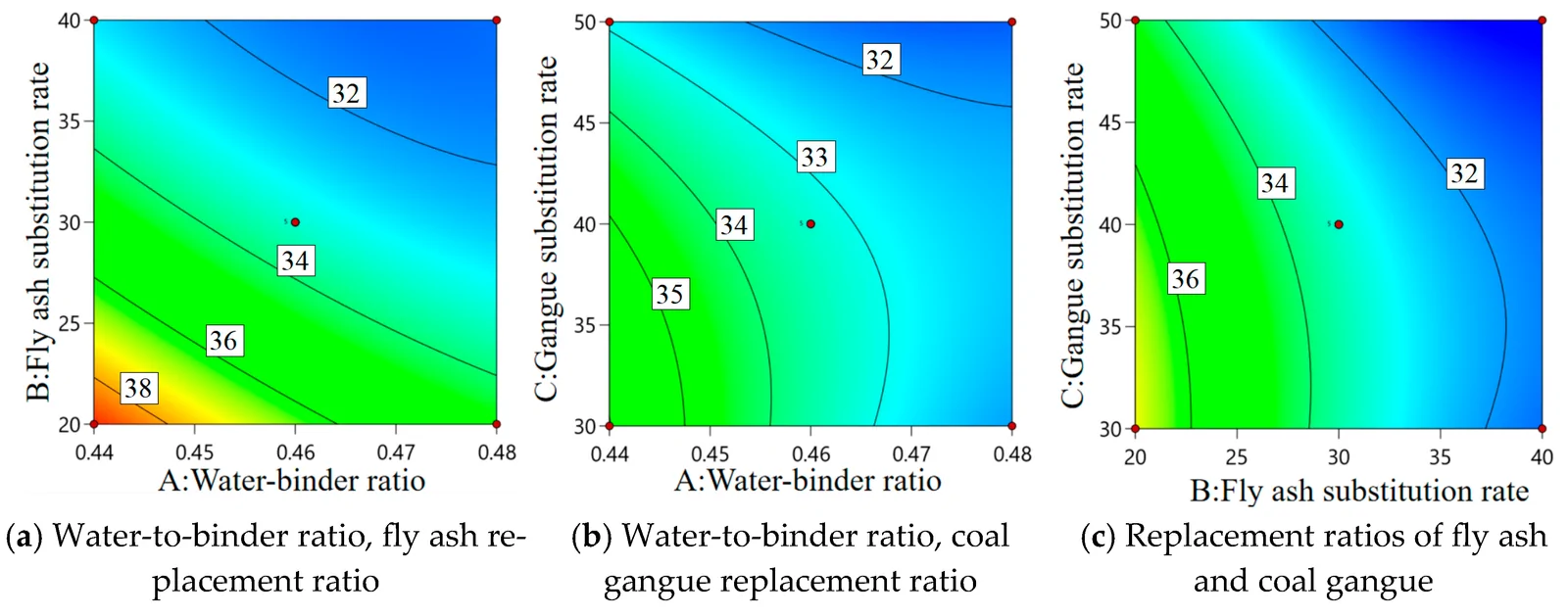
Effect of factor coupling on concrete strength.

**Figure 9:**
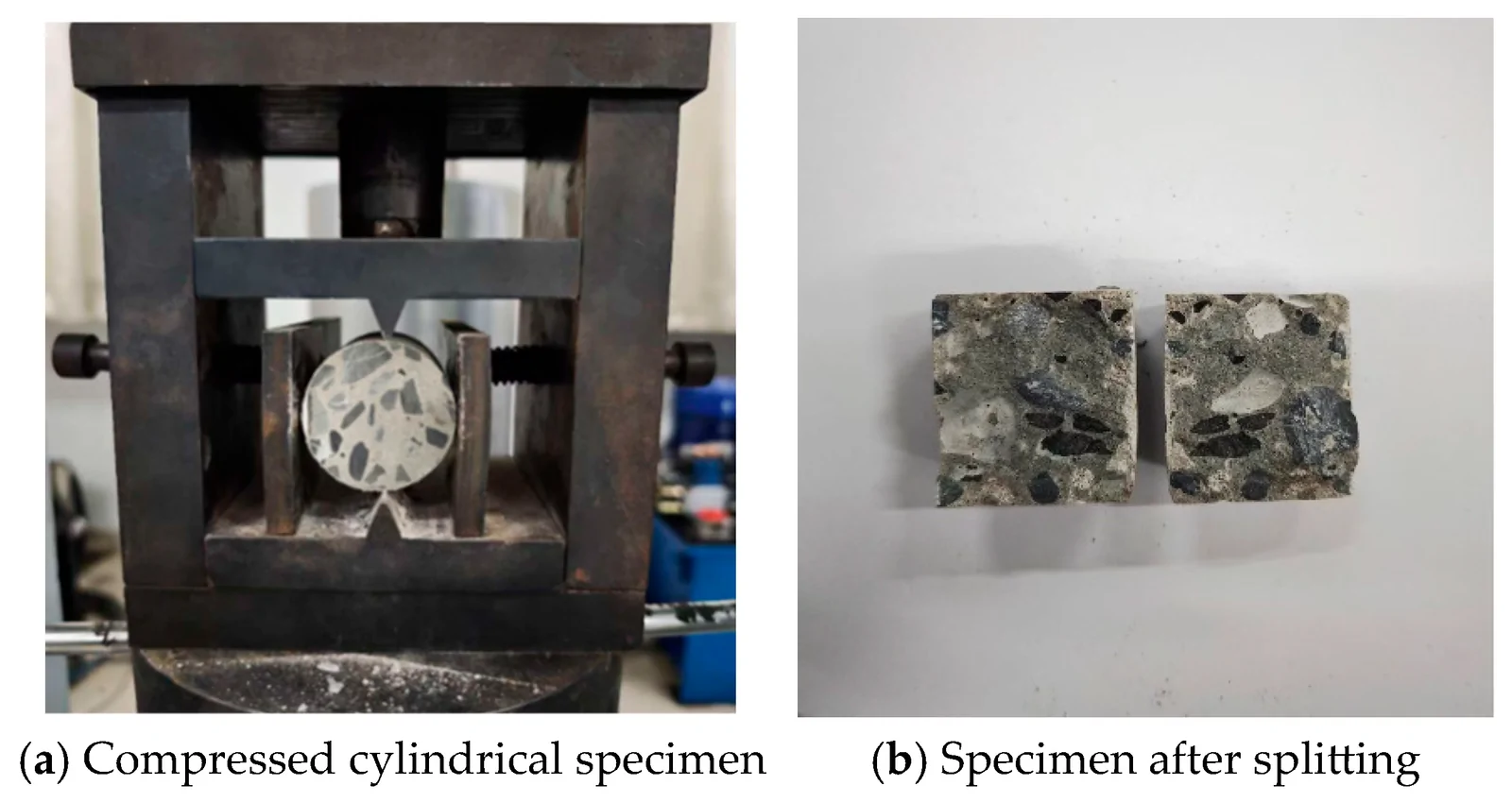
Brazilian splitting test process diagram.

**Figure 10:**
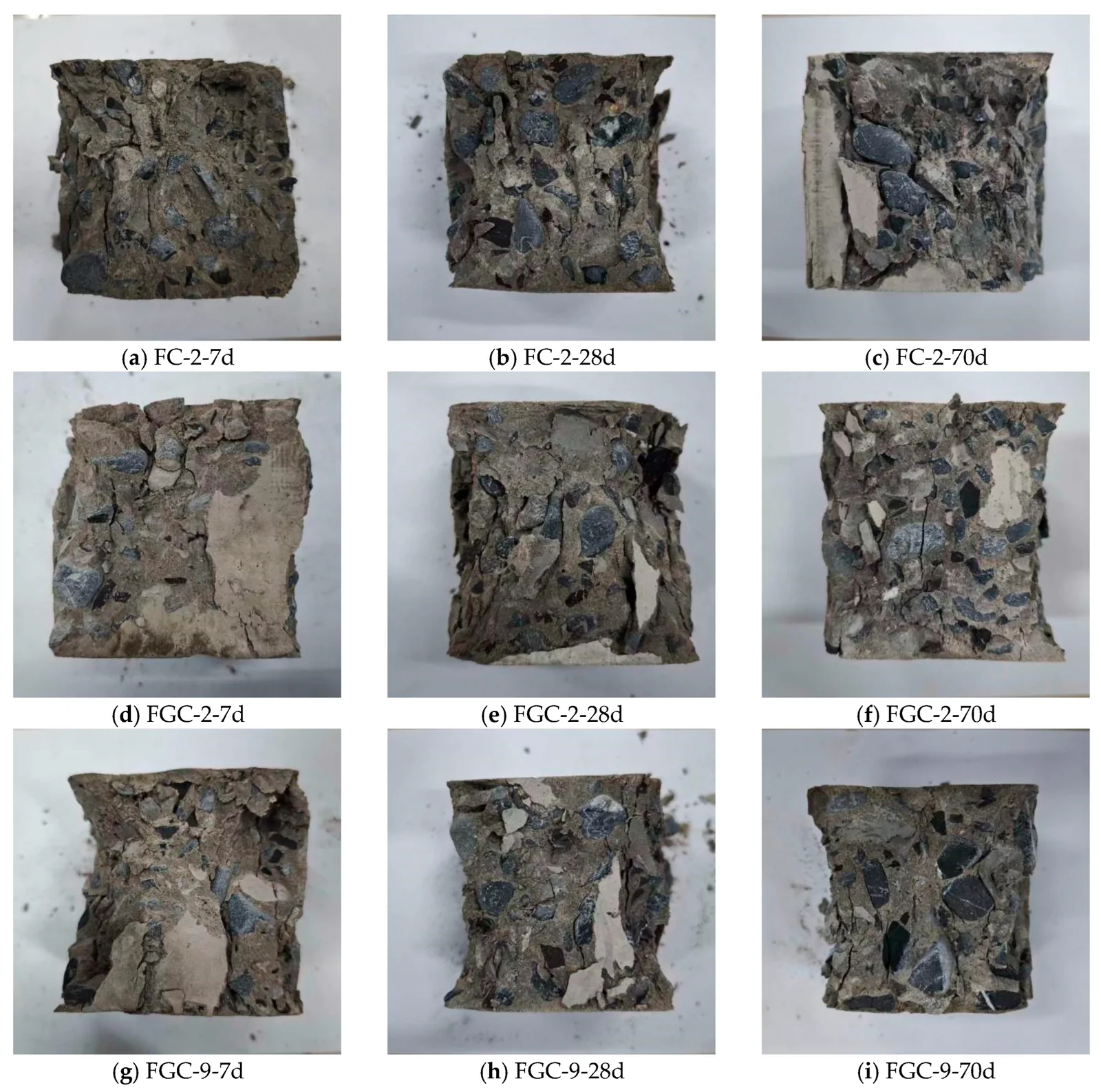

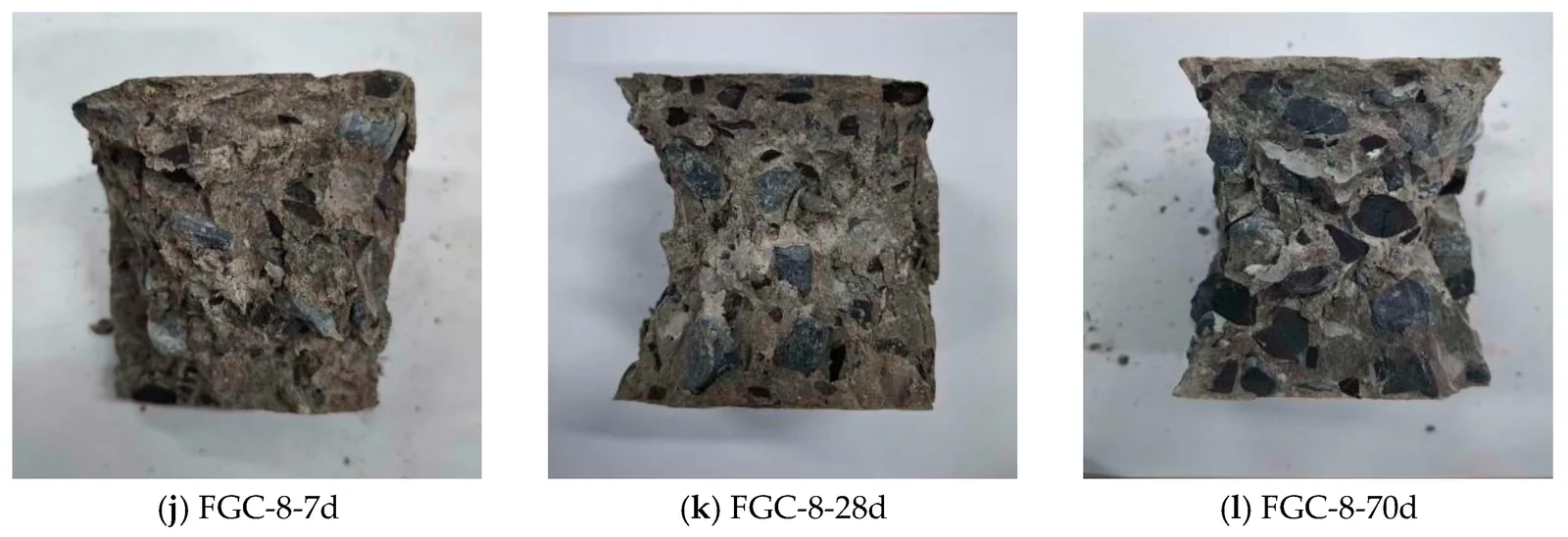
Compressed specimens with different mix proportions at various curing ages.

**Figure 11:**
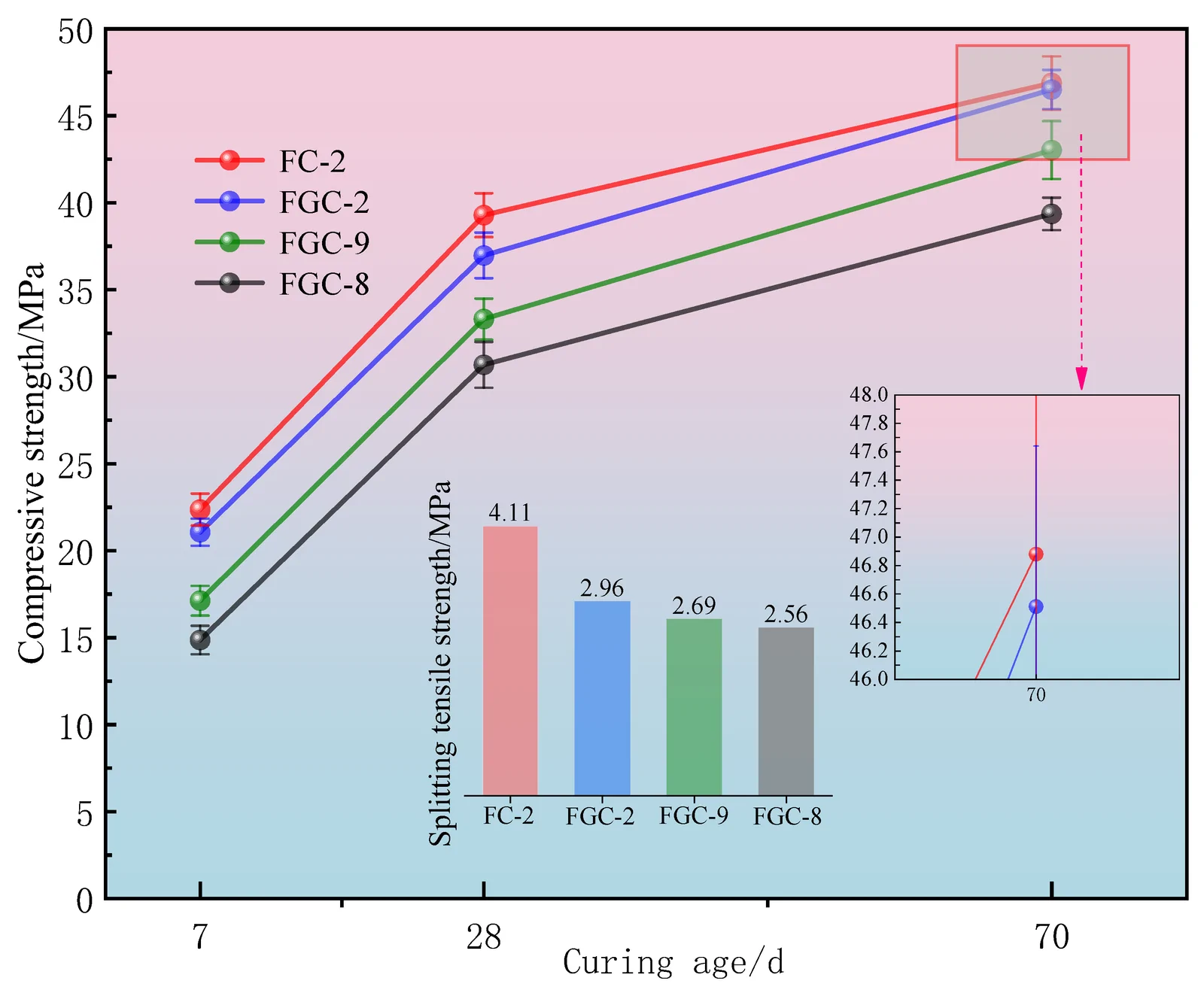
Chart of compressive strength variation trend and splitting tensile strength.

**Figure 12:**
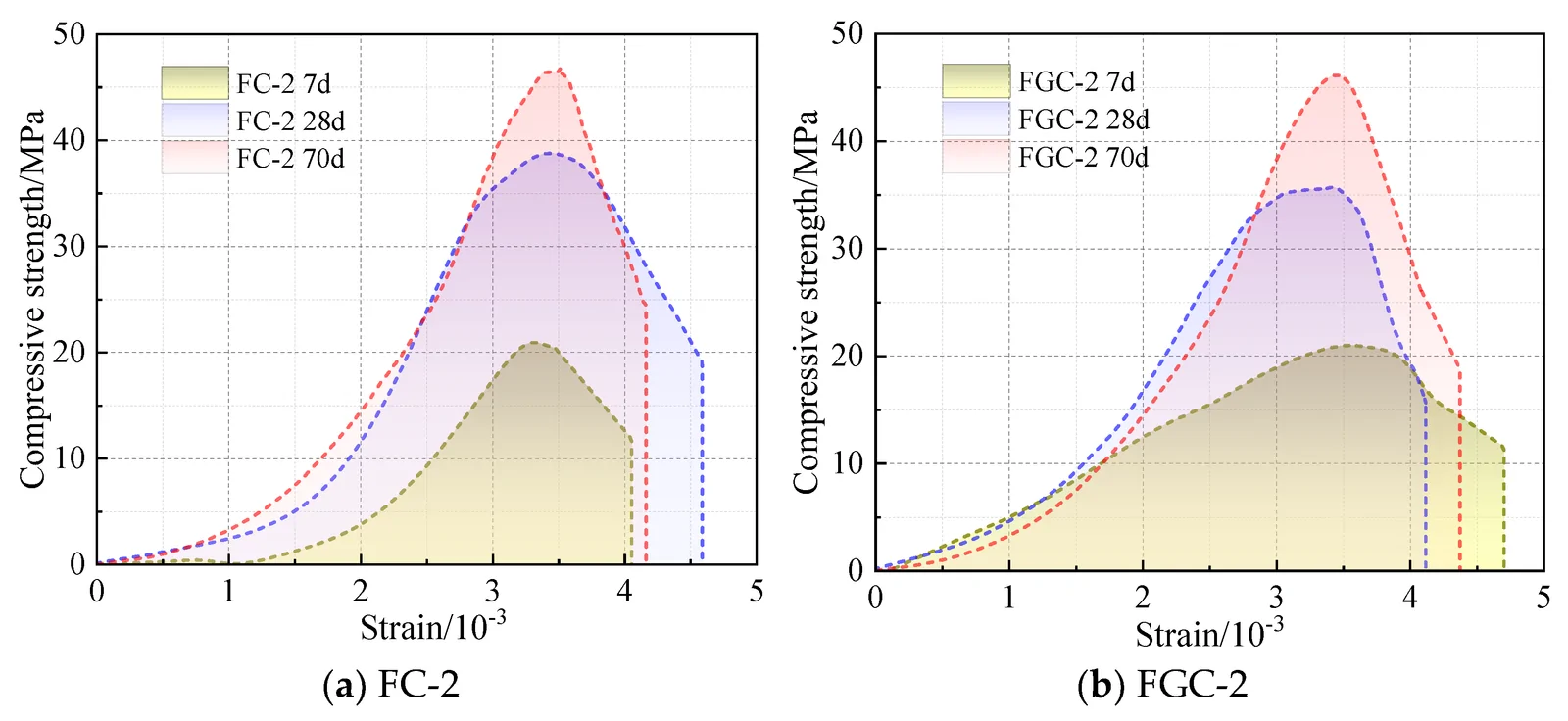

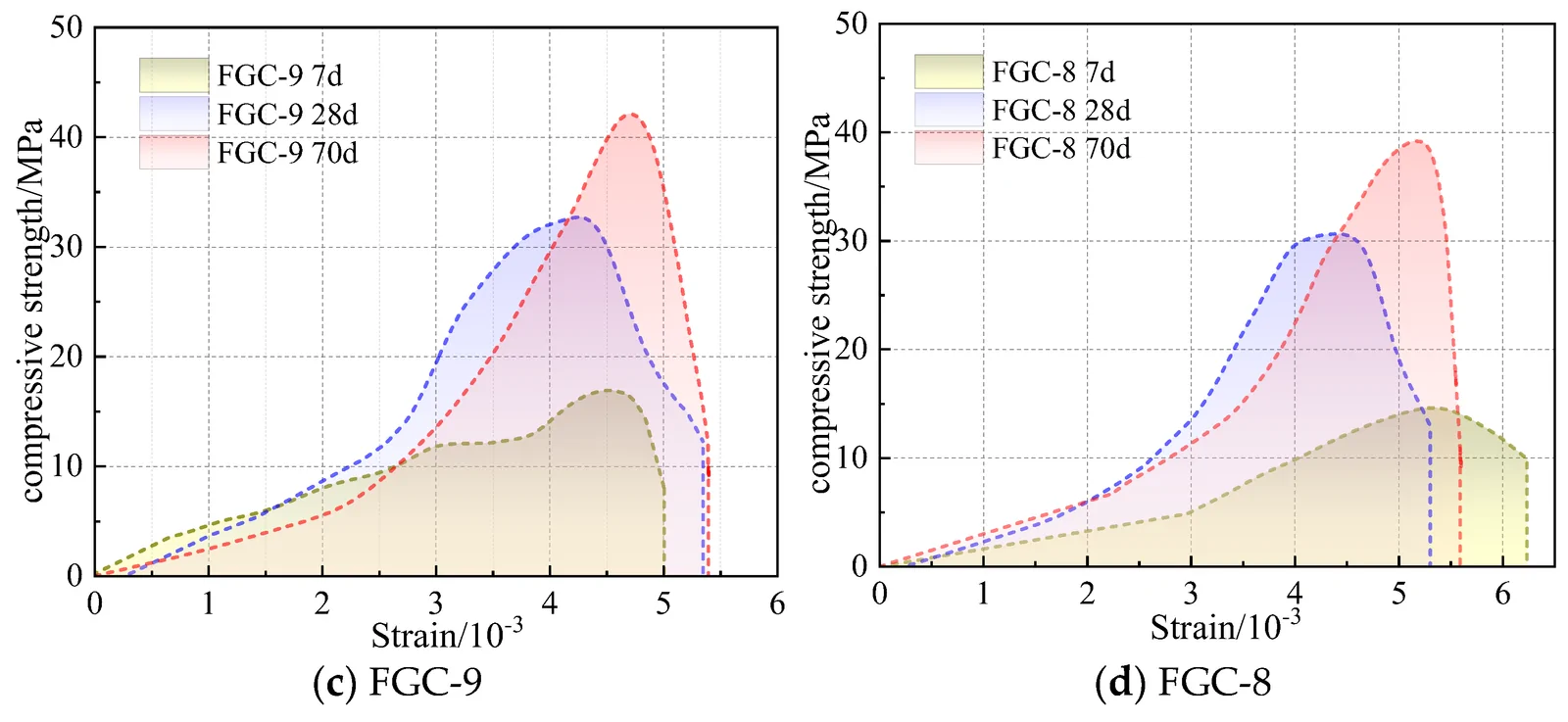
Stress–strain curves of different groups at various curing ages.

**Table 1:** Particle size distribution of coal gangue.

Nominal Particle Size Range (mm)	Graded Sieve Residue (%)	Cumulative Screening Residual (%)
>20	5.1	5.1
16–20	11.4	16.5
10–16	32.2	48.7
5–10	39.3	88.0
<5	9.3	97.3

**Table 2:** Comparison of physical properties between coal gangue and natural gravel.

Physical Properties	Coal Gangue	Natural Gravel
Grain composition (mm)	5–20	5–20
Crushing index (%)	19.8	10.3
Needle and flake particle content (%)	4.2	2.3
Density (kg/m^3^)	2340	2680
Moisture content (%)	3.1	1.3

**Table 3:** Main chemical composition of fly ash.

Chemical Composition	SiO_2_	Al_2_O_3_	Fe_2_O_3_	CaO	MgO	Loss on Ignition
Proportion of mass (%)	45.2	33.7	4.3	7.3	1.1	2.63

**Table 4:** Main chemical composition table of cement.

Chemical Composition	SiO_2_	Al_2_O_3_	Fe_2_O_3_	CaO	MgO	Loss on Ignition
Proportion of mass (%)	20.7	9.2	3.2	56.2	2.8	3.1

**Table 5:** Mix proportion of C30 fly ash concrete.

Number	Water–Binder Ratio	Fly Ash Substitution Rate/%	Material Usage (kg/m^3^)					
			Water	Fly Ash	Cement	Sand	Gravel	Admixture
FC-1	0.48	20	178	74.2	296.7	732	1098	1.48

**Table 6:** Mix proportion of coal gangue–fly ash concrete.

Number	Water–Binder Ratio	Fly Ash Substitution Rate/%	Coal Gangue Substitution Rate/%	Material Usage (kg/m^3^)						
				Water	Fly Ash	Cement	Sand	Gravel	Coal Gangue	Admixture
FGC-1	0.44	30	50	178	121.4	283.2	732	549.0	549.0	1.42
FGC-2	0.46	20	30	178	77.4	309.6	732	768.6	329.4	1.55
FGC-3	0.48	40	40	178	148.3	222.5	732	658.8	439.2	1.11
FGC-4	0.48	30	30	178	111.3	259.6	732	768.6	329.4	1.30
FGC-5	0.48	20	40	178	74.2	296.7	732	658.8	439.2	1.48
FGC-6	0.44	40	40	178	161.8	242.7	732	658.8	439.2	1.21
FGC-7	0.46	40	30	178	154.8	232.2	732	768.6	329.4	1.16
FGC-8	0.46	40	50	178	154.8	232.2	732	549	549	1.16
FGC-9	0.46	30	40	178	116.1	270.9	732	658.8	439.2	1.35
FGC-10	0.46	20	50	178	77.4	309.6	732	549	549	1.55
FGC-11	0.48	30	50	178	111.3	259.6	732	549	549	1.30
FGC-12	0.44	20	40	178	80.9	323.6	732	658.8	439.2	1.62
FGC-13	0.44	30	30	178	121.4	283.2	732	768.6	329.4	1.42
FGC-14	0.46	30	40	178	116.1	270.9	732	658.8	439.2	1.35
FGC-15	0.46	30	40	178	116.1	270.9	732	658.8	439.2	1.35
FGC-16	0.46	30	40	178	116.1	270.9	732	658.8	439.2	1.35
FGC-17	0.46	30	40	178	116.1	270.9	732	658.8	439.2	1.35

**Table 7:** Analysis of variance for response surface model.

Source	Square				Mean Square				F Value				*p* Value			
	$Y_{7d}$	$Y_{14d}$	$Y_{21d}$	$Y_{28d}$	$Y_{7d}$	$Y_{14d}$	$Y_{21d}$	$Y_{28d}$	$Y_{7d}$	$Y_{14d}$	$Y_{21d}$	$Y_{28d}$	$Y_{7d}$	$Y_{14d}$	$Y_{21d}$	$Y_{28d}$
Model	70.61	94.63	132.99	76.60	7.85	10.51	14.78	8.51	71.08	67.61	86.94	35.91	<0.0001	<0.0001	<0.0001	<0.0001
$X_{1}$	7.18	8.63	14.72	14.50	7.18	8.63	14.72	14.50	65.07	55.51	89.27	61.18	<0.0001	0.0001	<0.0001	0.0001
$X_{2}$	59.02	79.38	103.82	45.27	59.02	79.38	103.82	45.27	534.79	510.46	629.85	191.00	<0.0001	<0.0001	<0.0001	<0.0001
$X_{3}$	1.83	2.75	6.68	6.88	1.83	2.75	6.68	6.88	16.61	17.68	40.52	29.04	0.0047	0.0040	0.0004	0.0010
$X_{1}X_{2}$	0.6400	0.4356	2.10	2.79	0.6400	0.4356	2.10	2.79	5.80	2.80	12.75	11.77	0.0469	0.1381	0.0091	0.0110
$X_{1}X_{3}$	0.0784	0.1980	0.3422	1.65	0.0784	0.1980	0.3422	1.65	0.7104	1.27	2.08	6.97	0.4272	0.2963	0.1928	0.0534
$X_{2}X_{3}$	0.0306	0.1681	1.46	0.5852	0.0306	0.1681	1.46	0.5852	0.2775	1.08	8.88	2.47	0.6146	0.3331	0.5205	0.1601
$X_{1}^{2}$	0.8765	1.48	0.7650	0.8526	0.8765	1.48	0.7650	0.8526	7.94	9.53	4.64	3.60	0.0258	0.0176	0.0682	0.0997
$X_{2}^{2}$	0.7740	1.42	2.86	2.63	0.7740	1.42	2.86	2.63	7.01	9.13	17.33	11.09	0.0330	0.0193	0.0042	0.0126
$X_{3}^{2}$	0.0295	0.0188	0.0158	1.61	0.0295	0.0188	0.0158	1.61	0.2676	0.1206	0.0958	6.77	0.6209	0.7386	0.7659	0.0353
Residual value	0.7726	1.09	1.15	0.6194	0.1104	0.1555	0.1648	0.2370								
Misfit term	0.3862	0.5586	0.5285	0.6194	0.1287	0.1865	0.1762	0.2065	1.33	1.41	1.13	0.7944	0.3815	0.3627	0.4381	0.5574

**Table 8:** Mix proportion of floor concrete for roadway.

Number	Water–Binder Ratio	Fly Ash Substitution Rate/%	Coal Gangue Substitution Rate/%	Material Usage (kg/m^3^)						
				Water	Fly Ash	Cement	Sand	Coal Gangue	Gravel	Admixture
FC-2	0.46	20	0	178	77.4	309.6	732	0	1098	0.5%
FGC-2		20	30		77.4	309.6		329.4	768.8	
FGC-9		30	40		116.1	270.9		439.2	658.8	
FGC-8		40	50		154.8	232.2		549	549	

**Table 9:** Compressive strength at different curing ages and splitting tensile strength at long curing ages.

Number	7d Compressive Strength (MPa)	28d Compressive Strength (MPa)	70d Compressive Strength (MPa)	70d Splitting Tensile Strength (MPa)
FC-2	22.36	39.29	46.88	4.11
FGC-2	21.06	36.97	46.51	2.96
FGC-9	17.12	33.31	43.03	2.69
FGC-8	14.86	30.68	39.36	2.56

**Table 10:** Elastic moduli of concrete at different curing ages.

Curing Age/d	Elastic Moduli (GPa)			
	FC-2	FGC-2	FGC-9	FGC-8
7	16.26	7.57	5.76	4.49
28	26.13	21.79	18.65	17.16
70	31.24	28.35	20.91	19.74

## Data Availability

The original contributions presented in this study are included in the article. Further inquiries can be directed to the corresponding author.
